# Identifying sex-based disparities in porcine mitochondrial function

**DOI:** 10.1080/10495398.2025.2488068

**Published:** 2025-04-10

**Authors:** Hao Liu, Wenshu Shi, Xing Zhang, Xinmiao He, Xingbo Zhao

**Affiliations:** a Department of Gastroenterology, Sir Run Run Shaw Hospital, School of Medicine, Zhejiang University, Hangzhou, Zhejiang, China; b State Key Laboratory of Animal Biotech Breeding, China Agricultural University, Beijing, China; c Institute of Animal Husbandry, Heilongjiang Academy of Agricultural Sciences, Harbin, China

**Keywords:** Sex, porcine, mitochondrion, reactive oxygen species, oxygen consumption rate

## Abstract

In pigs, the effect of sex on production and reproductive traits has been largely reported, however, whether sex exerts its influence through regulating mitochondrial function is still unclear. In this study, we constructed 15 male cells and 15 female fibroblasts derived from 35-day and 50-day fetuses, newborn piglets and 1-year-old pigs to identify the sex effect on mitochondrial functions. Results indicated significant differences on cellular and molecular characteristics between male and female cells, including energy metabolic trait, mitochondrial DNA (mtDNA) replication and transcription, and mRNA expressions of mitochondrial biogenesis genes and mitoprotease genes. Referring to sex, males exhibited significantly higher oxygen consumption rate productions, levels of reactive oxygen species (ROS) and mtDNA copy numbers than those with females in muscle and ear fibroblasts. And the expressions of mtDNA, mitochondrial biogenesis genes (*POLG*, *PPARGC1A*, *TFAM* and *TWNK*) and *XPNPEP3* were higher in males than females in ear fibroblasts derived from 1-year-old adult pigs (EFA cells). While, the cell proliferation and expressions of genes related to ROS metabolism were not influenced by sex. The results highlight the effect of sex on mitochondrial function and gene expression, and provide important data for a comprehensive understanding of the mechanisms underlying sex regulation of energy metabolism-related traits in pigs.

## Introduction

Sex plays a significant role in shaping various economic traits in farm animals, particularly those associated with energy metabolism, such as feed efficiency, growth rates, body weight, reproduction and fat deposition. Studies reported that male pigs outperformed females in terms of growth traits (average daily gain, backfat depth and muscle depth), ratio efficiency traits (energy conversion ratio, Kleiber ratio and relative growth rate), as well as residual efficiency traits, including residual energy intake, residual daily gain, residual midtest metabolic weight and residual intake and gain.^1^ In various studies, it was observed that barrows consumed more feed, grew at a faster rate and possessed thicker backfat, lard and cheek weight, as well as a higher intramuscular fat content compared to females.^2–6^ On the other hand, females showed greater loin yield and higher linoleic acid content in subcutaneous fat.^4^^,^^5^ Additionally, females had a greater proportion of polyunsaturated fatty acid in backfat compared to barrows.^3^ Furthermore, females also exhibited greater fresh and trimmed ham yields compared to barrows.^4^ Besides, sex also has significant impacts on porcine metabolic processes. Barrows exhibited elevated levels of acylcarnitine and biogenic amines compared to gilts.^2^ Gilts had higher levels of several amino acids (Alanine, Arginine, Glycine, Histidine, Lysine, Serine, Threonine and Tryptophan), whereas castrated barrows exhibited higher levels of amino acid catabolism products, such as taurine, 2-aminoadipic acid and methionine sulfoxide. With the development of animal welfare policies in pig farming, it is possible that castration of boars may no longer be practiced in the future, resulting in an increasing number of uncastrated boars. Therefore, conducting in-depth research on the mechanisms of sex regulation in economic traits will be beneficial in advancing future pig breeding work.

Mitochondria, referred to as the powerhouse of cells, are double-membraned organelles that play a crucial role in energy production, metabolism and various cellular functions, including cell differentiation, hormone synthesis, apoptosis and fat deposition.^7^^,^^8^ They contain their own genome and ribosomes, enabling them to independently produce thirteen polypeptides.^9^ Given their maternal inheritance, it is hypothesized that mitochondrial function regulation differs between males and females, potentially regulating energy-related traits in animals. Overall, the relationship between sex and mitochondria is complex and multi-faceted. Understanding sex-specific differences in mitochondrial biology can provide valuable insights into the mechanisms underlying sex-related disparities in animal production traits. In this study, we constructed 15 male cells and 15 female cells derived from different aged fetuses/pigs, and analyzed the cell proliferation, reactive oxygen species (ROS) generation, replication and transcription of mitochondrial genome, aerobic respiration and expression of nuclear genes among cells, aimed to provide a deeper understanding on sex contributions to mitochondrial function.

## Methods

### Ethics approval and consent to participate

The guidelines of the experimental animal management of Heilongjiang Academy of Agricultural Sciences (HAAS) were followed throughout the study, and the animal study protocol was approved by the Institutional Review Board of the Institute of Animal Husbandry of HAAS, Heilongjiang, China (protocol code No. NKY-20140506, June 2004) (Supplementary Fig. S4).

### Cell isolation and culture

Primary fibroblasts (Table 1) were constructed using the collagenase digestion method. Briefly, fresh samples were collected and washed three times with phosphate-buffered saline and individually placed in dishes containing 5 mL collagenase digestion buffer (2 mg/mL collagenase in DMEM; Solarbio, China), diced and then incubated at 37 °C in a constant temperature shaker for 2 h. Afterward, the tissue digestive fluids were centrifuged, and fibroblasts were planked. Primary fibroblasts were cultured in DMEM (Gibco, California, USA) supplemented with 10% FBS (Gibco), 100 U/mL penicillin and 100 μg/mL streptomycin (Gibco), at 37 °C and 5% CO_2_/95% air. Fetal fibroblasts derived from 35-day-old fetuses were named as ‘FF cell’. Skin and muscle fibroblasts derived from 50-day-old fetuses were named as ‘SF cell’ and ‘MF cell’, respectively. Ear fibroblasts derived from newborn piglets and adult pigs were named as ‘EFN cell’ and ‘EFA cell’, respectively. Each group of fibroblasts contained six cell lines, of which three were male cells and rest were female cells, respectively.

**Table 1.** Primary fibroblasts constructed in this study.

Cell group	Cell source
FF cell (*n* = 6)	Fetal fibroblasts derived from 35-day-old fetuses
SF cell (*n* = 6)	Skin fibroblasts derived from 50-day-old fetuses
MF cell (*n* = 6)	Muscle fibroblasts derived from 50-day-old fetuses
EFN cell (*n* = 6)	Ear fibroblasts derived from newborn piglets
EFA cell (*n* = 6)	Ear fibroblasts derived from 1-year-old adult pigs

### Cell proliferation assay

The cell proliferation was analyzed by an Enhanced Cell Counting Kit-8 (CCK-8, Beyotime, C0039, China) according instructions. Nearly 2 × 10^3^ cells of each fibroblast were seeded in 96-well plates with 100 μL cell culture medium. Next, 10 μL of CCK-8 reagent was added to each well from 1 day to 7 days incubation, and then cells were incubated for 2 h at 37 °C with 5% CO_2_. Then, the absorbance at 450 nm was measured with reference wavelength at 650 nm using a microplate reader. Each cell contained at least three replicates and was tested three times.

### ROS measurement

The ROS level was detected by a ROS Assay Kit (Beyotime, S0033M, China) according instructions. Briefly, fibroblasts were plated at a density of 5 × 10^5^ cells per well in 6-well plates and allowed to adhere for 8 h under culture conditions. Subsequently, cells were incubated with 10 μM DCFH-DA for 20 min. Following three thorough washes with DMEM to remove excess probe, the fluorescence level of cell ROS was measured with excitation and emission settings at 488 and 525 nm, respectively, and expressed as arbitrary units.

### Quantification of mitochondrial DNA copy number and gene expression

Total RNA of fibroblasts was extracted by a Tissue/Cell RNA Rapid Extraction Kit (Aidlab, RN28, China) according to the manufacturer’s instructions, and the cDNA was synthesized using 1 μg RNA through a TRUEscript One Step qRT-PCR Kit (Aidlab, PC41, China). Besides, genomic DNAs of fibroblasts were extracted using a Tissue/Cell Genome DNA Extraction Kit (Aidlab, DN08, China). The relative mitochondrial DNA (mtDNA) copy number and mRNA expressions of genes (*12S rRNA*, *16S rRNA*, *ND6*, *COX1*, *CAT*, *ERO1A*, *ERO1B*, *SOD1*, *SOD2*, *SOD3*, *POLG*, *PPARGC1A*, *TFAM*, *TFB1M*, *TFB2M*, *TWNK*, *XPNPEP3*, *CLPP*, *PMPCA*, *ATP23*, *LONP1*, *BCS1L and OSGEPL1*) were measured by RT-qPCR with primers designed according to their gene sequences provided by NCBI or collected from our previous study^10^^,^^11^ (Supplememtary Table S1). *Beta-globin* and *GAPDH* were used as the internal control for detecting mtDNA copy number and gene expression, respectively. RT-qPCR was conducted on a qTOWER 2.2 instrument (ANALYTIKJENA, Germany) in a 10 μL volume containing 5 μL of 2 × SYBR Green qPCR Mix (Aidlab, PC33, China), 0.5 μL of each forward and reverse primer (10 μM), 1 μL of cDNA/DNA and 3 μL ddH_2_O according to the following program: 94 °C for 3 min; 94 °C for 10 s, 60 °C for 40 s for 40 cycles. The baseline adjustment method of the qPCRsoft software (ANALYTIKJENA, Germany) was used to determine the Ct of each reaction. A 2-fold dilution series of DNA/cDNA was included in each run to determine the PCR efficiency by constructing a relative standard curve using SYBR Green qPCR Mix (Supplementary Figs. S1–S3). All samples were amplified in triplicate. For data analysis, 2−^ΔΔ^*^ct^* method was used for calculating relative mtDNA copy number and mRNA levels of genes.

### Oxygen consumption rate detection

A Seahorse XFe96 analyzer (Seahorse Bioscience, Boston, USA) was used to determine the oxygen consumption rate (OCR) in fibroblasts. Primary fibroblasts were seeded in XF 96-well microplates at a density of 2 × 10^4^ cells per well and allowed to adhere for 8 h under culture conditions. Prior to analysis, the culture medium was replaced with XF assay medium. For respiratory analyses, cells were analyzed according to the procedures described in the Seahorse XF Cell Mito Stress Test kit (Agilent, 103015-100, California, USA). After baseline measurements of OCR, OCR was measured after sequentially adding to each well Oligomycin (2 μM), FCCP (0.5 μM) and Rotenone plus Antimycin A (0.5 μM of each). All data from XFe96 assays were collected using the Wave Desktop Software (Agilent, California, USA), and the measured values were normalized to the cell number of the primary fibroblasts plated on parallel plates.

### Statistical analysis

The mean values shown in figures represented at least three independent trials. The two-tailed unpaired Student t test was used for comparisons between two groups using the SAS software (SAS version 8.2). All results were presented as mean values ± standard error of the mean (SEM). A *p* value < .05 was considered statistically significant.

## Results

### Similar patterns of cell proliferation between female and male fibroblasts

**Figure 1.**
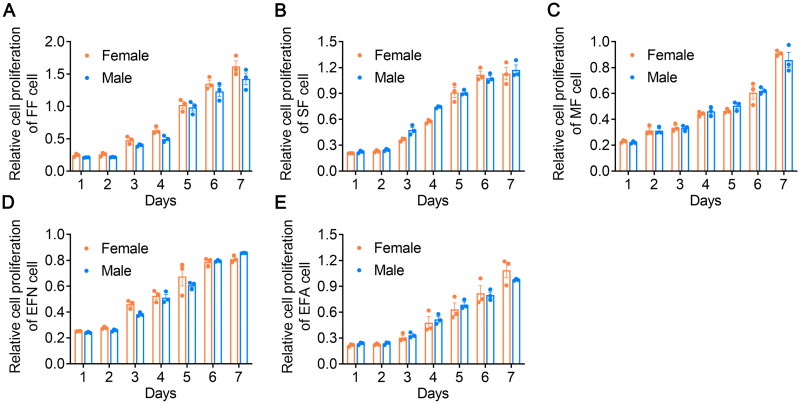
Cell proliferation assay between female and male fibroblasts. (A) Cell proliferation in FF cell. (B) Cell proliferation in SF cell. (C) Cell proliferation in MF cell. (D) Cell proliferation in EFN cell. (E) Cell proliferation in EFA cell. FF cell, fetal fibroblasts derived from 35-day-old fetuses; SF cell, skin fibroblasts derived from 50-day-old fetuses; MF cell, muscle fibroblasts derived from 50-day-old fetuses; EFN cell, ear fibroblasts derived from newborn piglets; EFA cell, ear fibroblasts derived from 1-year-old adult pigs. None of the differences were statistically significant by *t*-test.

Totally 30 porcine primary fibroblast lines were constructed, which were derived from different porcine tissues/organs and named as FF cell group, SF cell group, MF cell group, EFN cell group and EFA cell group (Table 1). Each group consisted of six individual primary cell lines, of which three cells were female, and the remaining three were male cells. To identify the effect of sex on cell proliferation, these five cell groups were analyzed using CCK-8 method. As shown in Fig. 1A, similar cell number were found between female and male FF cells at each detection time. Meanwhile, consistent results were also obtained in other fibroblasts, including SF cells, MF cells, EFN cells and EFA cells (Fig. 1B–E), which indicated sex contributed little to primary cell proliferation.

### The effect of sex on mtDNA replication and transcription

**Figure 2.**
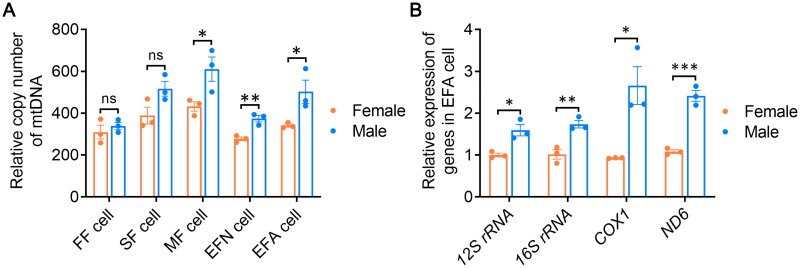
The mtDNA copy number and expression between female and male fibroblasts. (A) The relative copy number of mtDNA in fibroblasts, including FF cell, SF cell, MF cell, EFN cell and EFA cell. (B) The relative mRNA expressions of mitochondrial genes (*12S rRNA*, *16S rRNA*, *COX1* and *ND6*) in EFA cell. FF cell, fetal fibroblasts derived from 35-day-old fetuses; SF cell, skin fibroblasts derived from 50-day-old fetuses; MF cell, muscle fibroblasts derived from 50-day-old fetuses; EFN cell, ear fibroblasts derived from newborn piglets; EFA cell, ear fibroblasts derived from 1-year-old adult pigs; ns, no significance; *, *p* < .05; **, *p* < .01; ***, *p* < .001.

To elucidate the role of sex in the regulation of the replication and transcription of mtDNA, the mtDNA copy number and expressions were detected through RT-qPCR. As shown in Fig. 2A, the mtDNA copy number between female and male showed no significant differences in both FF cell group and SF cell group. Male cells exhibited higher contents of mtDNA than female cells in MF cell group, EFN cell group and EFA cell group (*p* < .05, Fig. 2A). Then, expressions of four genes encoded by mtDNA in EFA cell group were analyzed (Fig. 2B). The mRNA levels of *12S rRNA*, *16S rRNA*, *COX1* and *ND6* in male EFA cells were all significantly higher than those in female EFA cells (*p* < .05), indicating sex played a critical role in regulating activities of mitochondrial genome.

### The effect of sex on cell ROS accumulation

**Figure 3.**
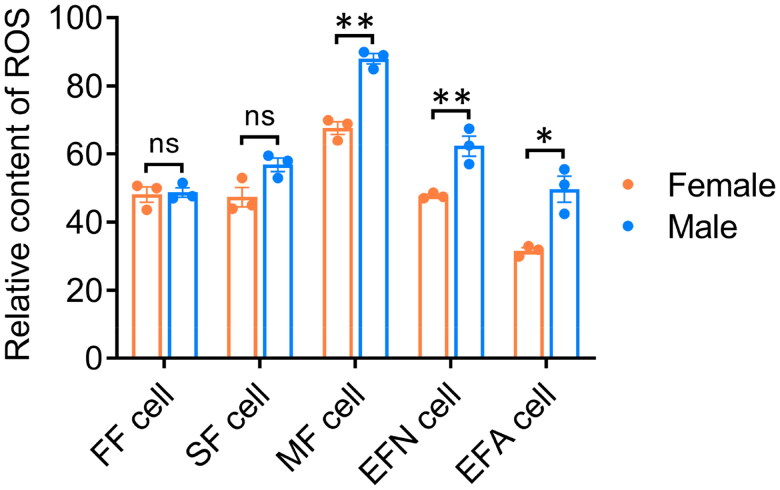
Cell reactive oxygen species (ROS) content in different sexes of fibroblasts. The relative ROS level between female and male fibroblasts. FF cell, fetal fibroblasts derived from 35-day-old fetuses; SF cell, skin fibroblasts derived from 50-day-old fetuses; MF cell, muscle fibroblasts derived from 50-day-old fetuses; EFN cell, ear fibroblasts derived from newborn piglets; EFA cell, ear fibroblasts derived from 1-year-old adult pigs; ns, no significance; *, *p* < .05; **, *p* < .01.

In order to identify the sex effect on cell ROS level, these five fibroblast groups were analyzed. As shown in Fig. 3, no significant changes were observed between female and male cells in either FF cell group or SF cell group; although the mean level of ROS of female cells in the SF group was slightly higher than that of MF cells (*p* > .05). Conversely, in MF, EFN and EFA cell groups, the ROS levels of female cells were all significantly higher compared to those of male cells.

### The sex effect on mitochondrial respiratory function

**Figure 4.**
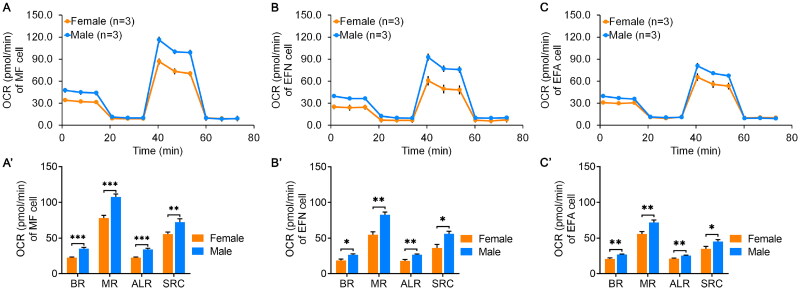
The oxygen consumption rate (OCR) between female and male fibroblasts. (A) The OCR assay in MF cell. (B) The OCR assay in EFN cell. (C) The OCR assay in EFA cell. (A’), (B’) and (C’) were OCR values of basal respiration (BR), maximal respiration (MR), ATP-linked respiration (ALR) and spare respiratory capacity (SRC) that analyzed using (A), (B) and (C) data, respectively. MF cell, muscle fibroblasts derived from 50-day-old fetuses; EFN cell, ear fibroblasts derived from newborn piglets; EFA cell, ear fibroblasts derived from 1-year-old adult pigs; *, *p* < .05; **, *p* < .01; ***, *p* < .001.

Given the observed sex-specific differences in cellular parameters (mtDNA copy number, mtDNA expression and ROS levels) across MF, EFN and EFA cells, we further evaluated their mitochondrial function by quantifying OCR through Seahorse. For the MF cell group, the basal respiration, maximal respiration, ATP-linked respiration and spare respiratory capacity of male cells were all extremely higher than those of female cells (*p* < .01, Fig. 4A and A’). In EFN and EFA cell groups, the differences of OCR values (basal respiration, maximal respiration, ATP-linked respiration and spare respiratory capacity) between male cells and female cells showed consistent patterns with the MF cell group (*p* < 0.05, Fig. 4B and C’).

### Gene expression patterns between male and female cells

**Figure 5.**
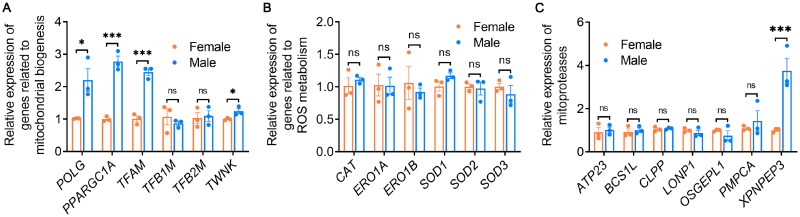
The mRNA expressions of genes between female and male fibroblasts. (A) The mRNA expressions of genes related to mitochondrial biogenesis in EFA cell. (B) The mRNA expressions of genes related to ROS metabolism in EFA cell. (C) The mRNA expressions of mitoproteases in EFA cell. EFA cell, ear fibroblasts derived from 1-year-old adult pigs; ns, no significance; *, *p* < .05; **, *p* < .01; ***, *p* < .001.

To investigate the potential role of sex-related differences in the replication and expression of mitochondrial genome and OCR levels, we examined the mRNA levels of mitochondria biogenesis-related genes derived from the nuclear genome. As shown in Fig. 5A, the expressions of *POLG*, *PPARGC1A*, *TFAM* and *TWNK* in male EFA cells were all significantly higher than those in female cells. Among them, *POLG*, *PPARGC1A* and *TFAM* showed over twofold differences in expressions between female and male cells. In addition, expression patterns of *TFB1M* and *TFB2M* were similar between male and female EFA cells. Besides, we further examined the expressions of nuclear genes involved in ROS metabolism. It was interesting that the mRNA levels of six genes (*CAT*, *ERO1A*, *ERO1B*, *SOD1*, *SOD2* and *SOD3*) showed no significant differences between male and female EFA cells (Fig. 5B). Mitoproteases encoded by nuclear genes play a crucial role in maintaining mtDNA stability, regulating the assembly of mitochondrial complexes and clearing ROS within mitochondria. Therefore, we examined whether the expressions of seven mitoproteases (*ATP23*, *BCS1L*, *CLPP*, *LONP1*, *OSGEPL1*, *PMPCA* and *XPNPEP3*) were affected by sex. It was to note that the level of *XPNPEP3* in male EFA cells was over 3.5-fold higher than that in female cells (Fig. 5C).

## Discussion

Sex has a significant influence on various traits in pigs, especially those relevant for economic purposes. Traditionally, male pigs have been castrated to prevent boar taint in pork production systems.^12^^,^^13^ However, following the EU agreement to abolish surgical castration of piglets by 2018 (European Declaration on alternatives to surgical castration of pigs in 2010), there is a growing trend toward rearing intact males, which may impact minced carcass chemical composition and meat quality.^14^ Therefore, it is vital to conduct comparative studies involving different sex types. As body composition changes throughout growth, investigating the effects of sex at various stages of pig development contributes to understanding whether the variations between sex types vary based on the type of fibroblasts. Thus, in this study, we established a total of five fibroblast groups derived from fetuses or pigs of different ages, namely FF cell, SF cell, MF cell, EFN cell and EFA cell, respectively (Table 1). Male pigs generally exhibit higher average daily gain and relative growth rate compared to females.^1^ Additionally, barrows also grow faster and consume more feed than female pigs.^6^ Cell proliferation is mainly regulated by several factors, including nutrient availability, growth factors, hormones (such as sex and growth hormones), extracellular matrix, cell density and culture environment. In this study, the cell proliferation assay was first performed. Interestingly, among these five fibroblast groups, no significant difference was observed in cell proliferation levels between male and female cells within each group (*p* > .05, Fig. 1). This could be because, during the cell culture process, both male and female fibroblasts received the same nutrients from the culture medium, which may minimize the effect of genes on the sex chromosomes. Additionally, the absence of differential regulation by sex hormones and growth hormones could also contribute to the similar proliferation rates observed between male and female cells under normal culture conditions.^15^^,^^16^

To analyze the impact of sex on porcine mitochondria, we first measured the content of mtDNA in each cell group. We found that in FF cell group and SF cell group, the copy number of mtDNA was similar between male and female cells. However, in MF cell group, EFN cell group and EFA cell group, the relative mtDNA copies were significantly higher in male cells than in females (Fig. 2). Additionally, the relative levels of cellular ROS showed consistent results (Fig. 3). These findings suggested that the effect of sex on mtDNA copy number and cellular ROS levels was regulated by the cell source. Studies have indicated that in mouse and human cardiac cells, females exhibit lower mtDNA content and ROS production compared to males,^17^^,^^18^ which is consistent with our findings in pigs (Figs. 2 and 3). It suggested that the phenomenon of cells from male individuals after birth exhibited more mtDNA copies and higher ROS contents than females was relatively conserved across species.

The level of cellular ROS content is regulated by multiple factors. Studies have shown that the mitochondrial ROS primarily originates from the activity of ETC complexes I and II.^19^ While the generation of ROS can lead to DNA damage and promote cell ageing.^20^^,^^21^ In this study, we found that both the expression of *ND6* gene of mitochondrial complex I (Fig. 2B) and the cellular OCR level (Fig. 4) were significantly higher in male cells compared to females, suggesting that ETC complexes were more active in male cells, which might be one of primary reasons for the relatively higher ROS content. Additionally, we also examined the expression of genes involved in ROS metabolism process. We found that in EFA cells, the expressions of six genes (*CAT*, *ERO1A*, *ERO1B*, *SOD1*, *SOD2* and *SOD3*) were similar between males and females (Fig. 5B), indicating that under the normal culture condition, the ROS level in male cells still remained within the normal range and was not sufficient to active the ROS metabolism pathway, although it was relatively higher compared to females.

The transcription and translation processes of genes in mtDNA are both carried out within the mitochondrial matrix. In this study, we found that the expression levels of *COX1* and *ND6* were significantly higher in EFA male cells compared to females (Fig. 2B). Consistent results were also obtained for the detection of *12S rRNA* and *16S rRNA*, suggesting that in adult male cells, the expression activity of mitochondrial genes was higher compared to female cells. Previous studies reported that several proteins played important regulatory roles in the mitochondrial biogenesis process, such as POLG, PPARGC1A and TFAM.^22–24^ In this study, we found that male cells possessed higher mRNA levels of *POLG*, *PPARGC1A*, *TFAM* and *TWNK* compared to female EFA cells (Fig. 5A). Importantly, the expression differences of *POLG*, *PPARGC1A* and *TFAM* between the two sexes were over twofold, suggesting that the high expression of these three genes in males was an important factor to elevate mtDNA replication and expression. However, further study is necessary to explore mechanisms underlying the sex regulation of gene expression related to mitochondrial biogenesis.

Based on the differential levels of mtDNA copy number and transcription between males and females, we further analyzed the impact of these differences on cellular OCR. Interestingly, in MF, EFN, EFA cell groups, OCR values (basal respiration, maximal respiration, ATP production and spare respiratory capacity) in male cells were generally higher than those in females (Fig. 4), which were consistent with the results of mtDNA replication, transcription and mitochondrial biogenesis between males and females (Figs. 2 and 5). These findings suggested that, in general, under the ample nutrition condition, male cells in postnatal individuals exhibited higher levels of aerobic respiration compared to female cells.

Mitoproteases are a class of proteases that exist within mitochondria and play crucial regulatory roles in mitochondrial function. They are involved in various processes such as mitochondrial protein transport, processing and degradation, assembly of mitochondrial complexes, mitochondrial fission and fusion, lipid synthesis and stability of mtDNA. In this study, we also examined expressions of seven mitoprotease genes. The results revealed that the expression of *XPNPEP3* in EFA male cells was significantly higher than in females (Fig. 5C). X-Pro aminopeptidase 3 (XPNPEP3) belongs to the family of X-pro-aminopeptidases, which can remove the N-terminal amino acid from some MPP-processed substrates with a proline residue in the penultimate position.^25^ Studies have shown that the absence of Icp55 (a homolog of XPNPEP3) in yeast cells increases chronological lifespan and oxidative stress resistance, while decreasing mitochondrial oxygen consumption and ATP synthase complex assembly.^26^ Based on the previous reports and results of this study, we speculated that XPNPEP3 was involved in the regulation process of in the regulation of sex-mediated cellular energy metabolism processes.

## Conclusion

With the ongoing promotion of animal welfare policies, there will be a gradual increase in the population of intact male pigs. Studies on the sex regulation of energy metabolism-related traits are crucial for pig breeding. This research yielded interesting findings: male pig cells demonstrated higher mtDNA copy numbers and transcriptional activity, as well as increased levels of ROS, OCR and expression of genes associated with mitochondrial biogenesis and mitoprotease. Besides, the effects of sex on cellular functions were also influenced by the age and tissue of pigs. In summary, this study explored the variations in energy metabolism among cells obtained from pigs of different ages, tissues and sexes, providing a theoretical foundation for improving animal welfare and breeding.

## Supplementary Material

Figure S3.tif

Figure S1.tif

Figure S2.tif

Table_S1 clean.docx

Figure S4.pdf

## Data Availability

All data generated or analyzed during this study are included in this published article [and its supplementary information files].
