# Supplementary figures and images for: Identifying sex-based disparities in porcine mitochondrial function
Source: Anim Biotechnol. 2025 Apr 10;36(1):2488068. doi: 10.1080/10495398.2025.2488068 (PMC12674342; doi:10.1080/10495398.2025.2488068)

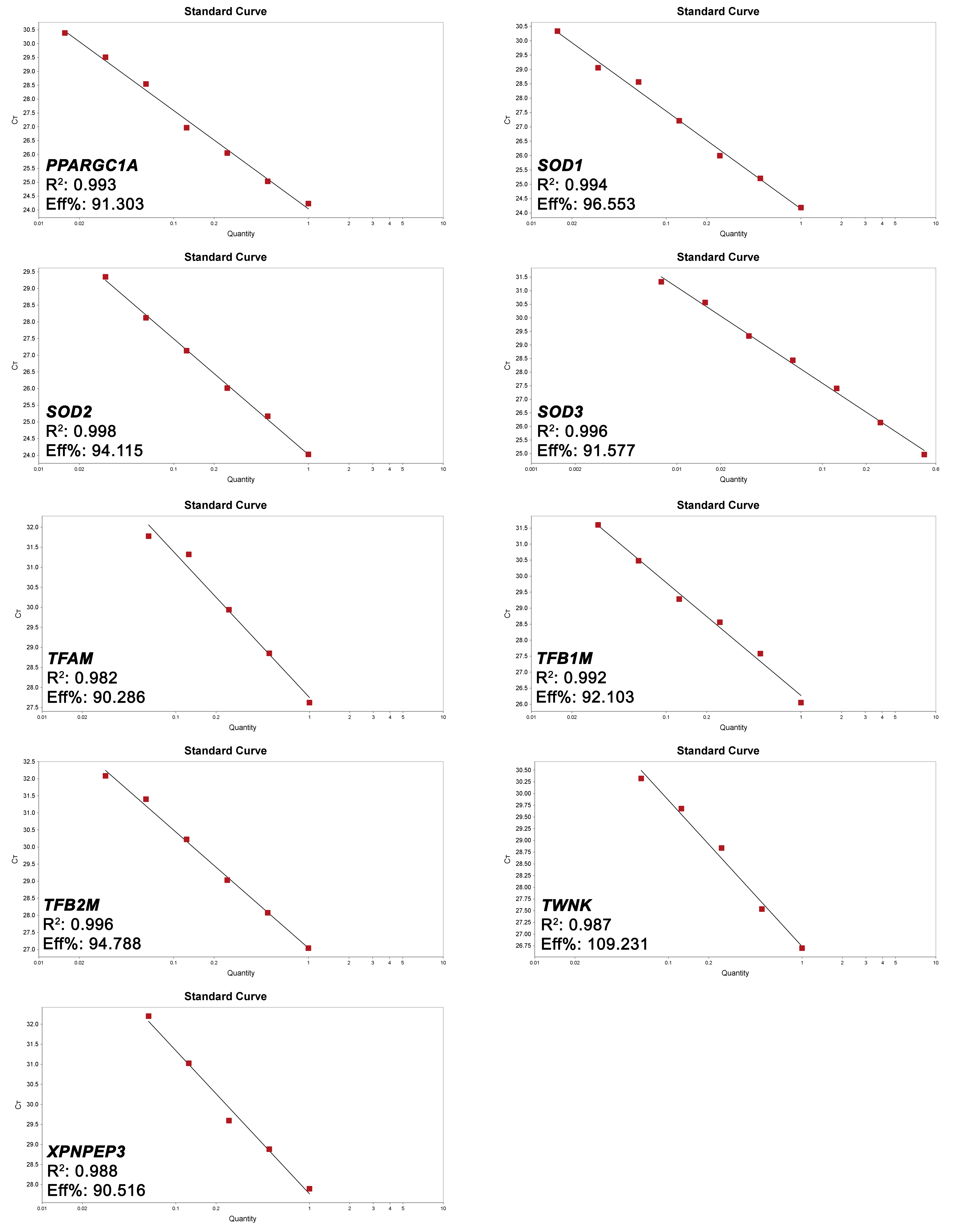

Supplement: Figure S3.tif [file LABT_A_2488068_SM2601.tif]

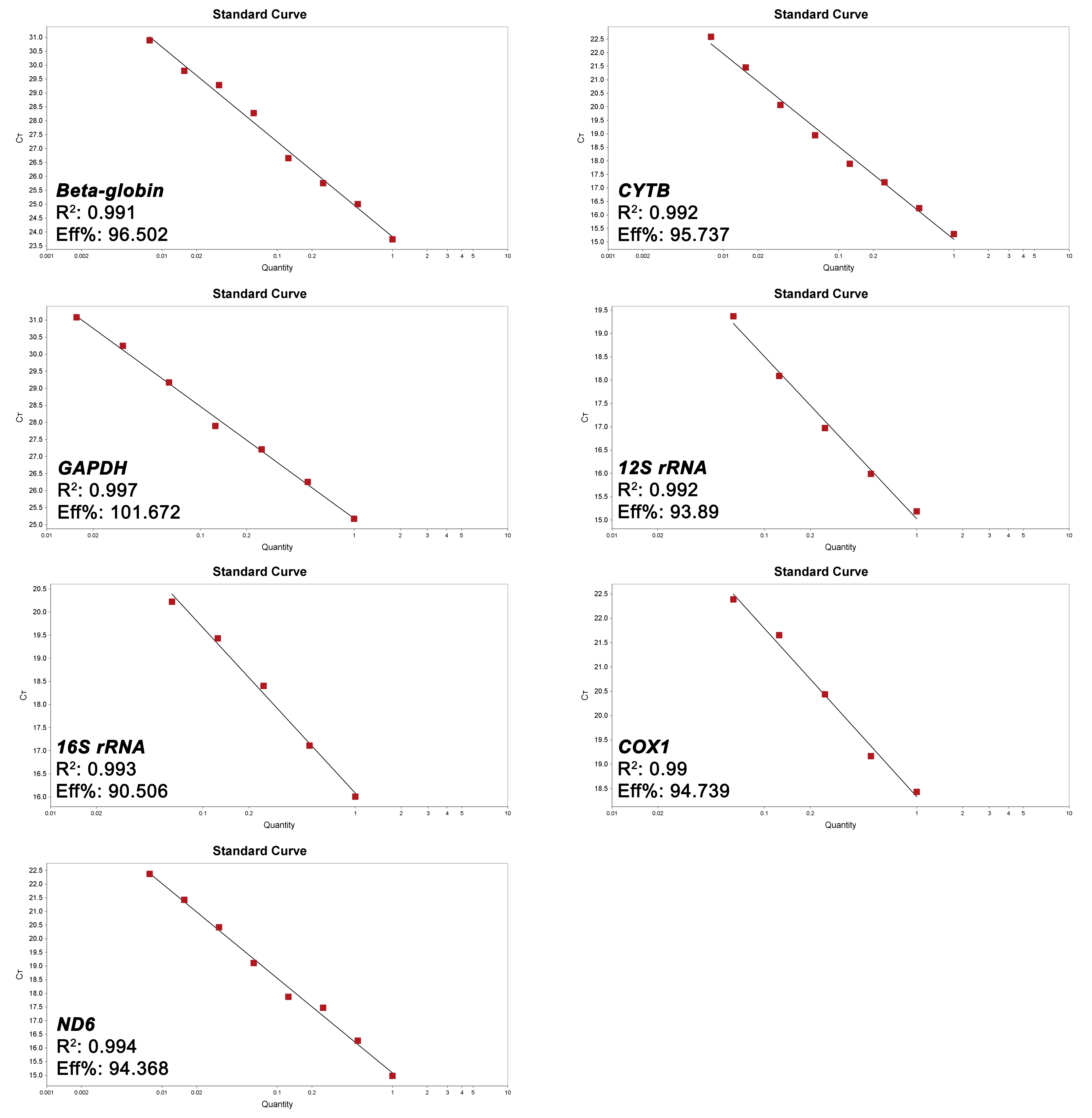

Supplement: Figure S1.tif [file LABT_A_2488068_SM2600.tif]

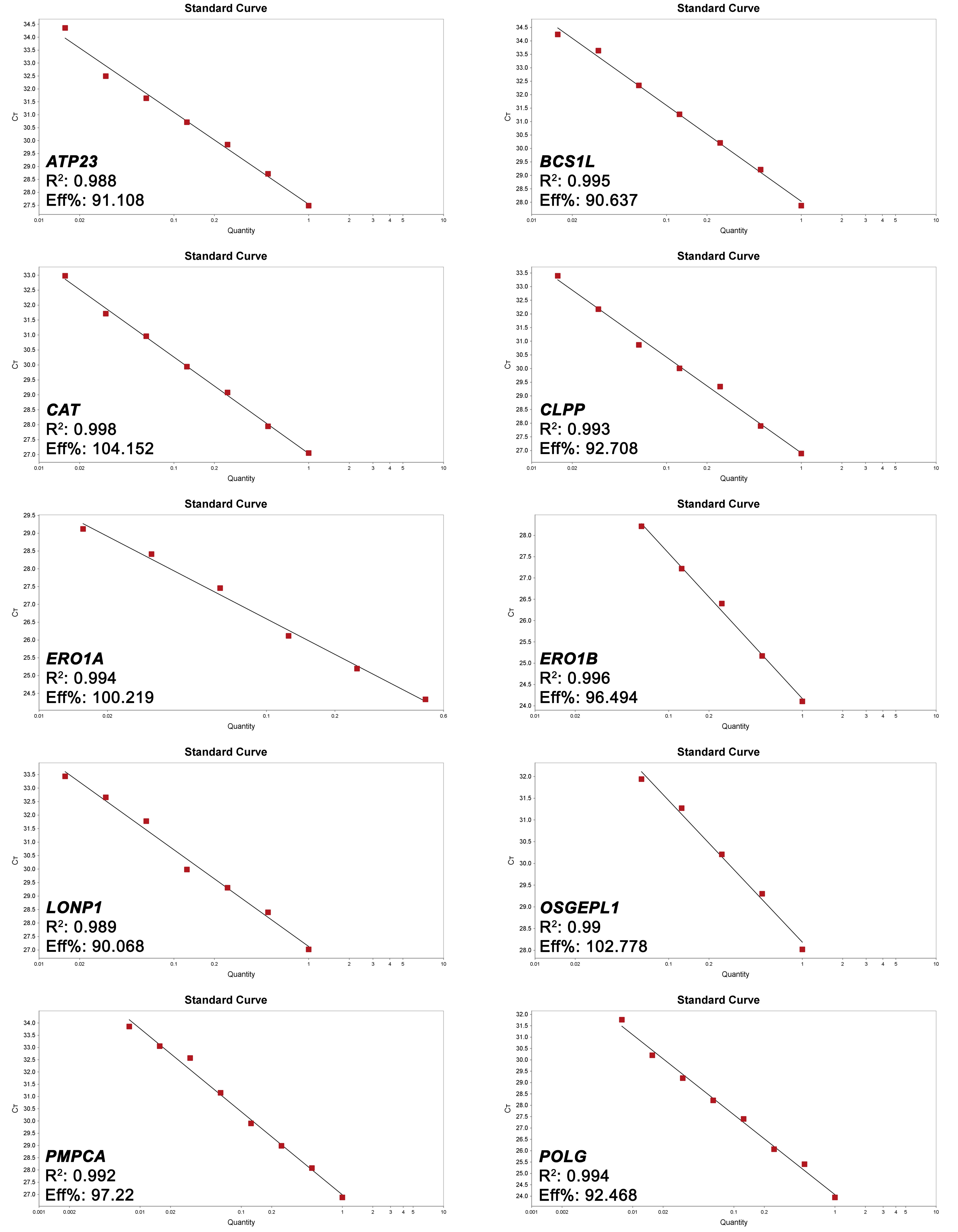

Supplement: Figure S2.tif [file LABT_A_2488068_SM2599.tif]
