# Supplementary material for: Identifying sex-based disparities in porcine mitochondrial function
Source: Anim Biotechnol. 2025 Apr 10;36(1):2488068. doi: 10.1080/10495398.2025.2488068 (PMC12674342; doi:10.1080/10495398.2025.2488068)
Supplement: Table_S1 clean.docx [file LABT_A_2488068_SM2598.docx]

Table S1. Primers used in this study.

| Target | Sequence (5'→3') |
| --- | --- |
| mtDNA copy number |  |
| *Beta-globin* | F: AGCCAGCAGCCACCTACATT |
|  | R: CCACCAACTTCGTCCACATTCA |
| *CYTB* | F: ACCTACTAGGAGACCCAGACAACT |
|  | R: TGAACGTAGAATAGCGTAGGCGAA |
| mRNA expression |  |
| *GAPDH* | F: ATTCCACCCACGGCAAGTTC |
|  | R: GTTCACGCCCATCACAAACA |
| *12S rRNA* | F: TACCGCCATCTTCAGCAA |
|  | R: CCCATTTCTTTCCAACCC |
| *16S rRNA* | F: GAAGCCTTTCTCCTCGCACA |
|  | R: AAACTGGTGTCACTGGGCAG |
| *ND6* | F: CTACTGCTATGGCTACTGAGATGTATCC |
|  | R: CTTCCTCTTCCTTCAACGCA |
| *COX1* | F: ATAGGAGCAGTGTTCGCCAT |
|  | R: TGCGTCAGGATAATCGGAGT |
| *CAT* | F: ACGTTCTGTAAGGCTAGTCGGA |
|  | R: GGTCAGGATATCAGGTTTCTGCG |
| *ERO1A* | F: GTCAAGCCTTGTCAGTCGGA |
|  | R: CCTTCTGCGTTTCCTCACTCA |
| *ERO1B* | F: AAAAGACTGTCATGTGGAGCCT |
|  | R: GCTGGCGATCTCTCATCATCAA |
| *SOD1* | F: AAGGATCAAGAGAGGCACGTTG |
|  | R: CTTCCAGCATTTCCCGTCTTTG |
| *SOD2* | F: ATCAACGCCCAGATCATGCAG |
|  | R: CCTGAGCTGTAACATCACCCTT |
| *SOD3* | F: CTCTGTGCTTACCTGCTCCTGG |
|  | R: AGATCTCCGTCACTTTGGCCT |
| *POLG* | F: TGACTCTCCTATTGGTCGCACT |
|  | R: CCTTCCACCCAACAAATCCTCA |
| *PPARGC1A* | F: GCTATGGTTTCATTACCTACCG |
|  | R: ATCCTCAGCCAGGGAACAT |
| *TFAM* | F: TGTGCGGTTTGTGGAAGT |
|  | R: ACCTGCCAGTCTGCCCTAT |
| *TFB1M* | F: CGAGGGCTTGGAATGTTA |
|  | R: CGTGTGCCTGAGTTCTTCT |
| *TFB2M* | F: GCAAGGAGGAAGGATGTT |
|  | R: CAAGTAATGCTCGTGTCAGG |
| *TWNK* | F: TAAGGCTAAAGCTGAGACGCTG |
|  | R: CGCTATGACCTCGGAAGCAAAA |
| *XPNPEP3* | F: TGGTACCGAAACTGAAAGCTGA |
|  | R: CACCATTTCCCCATCCTGTGA |
| *CLPP* | F: ATCATGATCCACCAGCCCTCTG |
|  | R: TCCATGGCCGACTCAATCAC |
| *PMPCA* | F: CGTTCTCTGGGGTGACTTTACA |
|  | R: GCTTTATTCCACTGACTGGCAC |
| *ATP23* | F: AGAATTGGCTCCTTGATGCTACA |
|  | R: GTTTGACATATGGATTCAGGCG |
| *LONP1* | F: TGAGAGCCTGGACGAGATCTAT |
|  | R: TGTCTGTTGATGTGAACCCTCC |
| *BCS1L* | F: ATCTGGTGGGAAACAGGTCTTG |
|  | R: CCAAAGTAGGGGTTGTCCTTCA |
| *OSGEPL1* | F: CGACGGATTCGGGCTTTCT |
|  | R: TTACTGAACAGATAATTCCTTCCTTG |
