# Supplementary material for: Identifying sex-based disparities in porcine mitochondrial function
Source: Anim Biotechnol. 2025 Apr 10;36(1):2488068. doi: 10.1080/10495398.2025.2488068 (PMC12674342; doi:10.1080/10495398.2025.2488068)
Supplement: Figure S4.pdf [file LABT_A_2488068_SM2597.pdf]

黑龙江省农业科学院畜牧研究所
实验动物福利与伦理审查表

项目名称	民猪抗猪流行性腹泻病毒感染及其遗传机制研究		
项目负责人	何鑫淼	联系电话	13896777337
动物品种	猪	实验起止日期	2024.1.1-2025.12.31
实验地点	黑龙江省农业科学院畜牧研究所		
实验类别	☑涉及动物实验<br>☑涉及组织或细胞实验（含人体和动物组织细胞）		
实验方法	本研究拟开展不同品种猪在抗猪流行性腹泻病毒的感染机制上的研究，主要利用民猪、巴克夏猪。巴民杂交猪和大白猪等，进行攻毒保护研究。包括对其生长性能，组织病毒拷贝数，不同免疫细胞的活性，抗感染分子表达、转录组和表观遗传进行研究。上述研究各个品种猪各选用 60 头。		
项目负责人承诺	本人承诺，在项目实施过程中按照国家标准实验动物福利伦理审查指南（GB/T35892-2018）的有关要求开展研究，最大程度减轻动物的疼痛、痛苦和紧张，保护动物的权益。<br>项目负责人签字：何鑫淼		
审查意见	该研究实验设计和方案充分考虑了安全性和公平性，充分考虑了实验动物替代、减少和优化，最大程度保护动物的权益。<br>（盖章）<br>2023 年 12 月 1 日		

Animal Husbandry Research Institute, Heilongjiang Academy of Agricultural Sciences

Application Form of Laboratory Animal Welfare and Ethics Review

<table>
<tr><td>Protocol Title</td><td colspan="3">Research on the Resistance of Min Pigs to Porcine Epidemic Diarrhea Virus Infection and Its Genetic Mechanisms</td></tr>
<tr><td>Principal Investigator</td><td>Xinmiao He</td><td>Tel</td><td>+86-13896777337</td></tr>
<tr><td>Animal Species</td><td>Pig</td><td>Proposed Period of Animal Experiment</td><td>1/1/2024-12/31/2025</td></tr>
<tr><td>Animal Experiment Site</td><td colspan="3">Animal Husbandry Research Institute, Heilongjiang Academy of Agricultural Sciences</td></tr>
<tr><td>Study Type</td><td colspan="3">☑ Animal experiments
☑ Tissue or cell experiments (including human and animal tissue/cell studies)</td></tr>
<tr><td>Experimental Methods</td><td colspan="3">This study aims to investigate the infection resistance mechanisms of different pig breeds against Porcine Epidemic Diarrhea Virus (PEDV), primarily utilizing Min pigs, Berkshire pigs and other pig breeds. The study involves challenging pigs with PEDV to analyze its effects on growth performance, viral copy numbers in tissues, activity of diverse immune cell populations, expression of anti-infection molecules, as well as transcriptomic and epigenetic regulatory mechanisms. For each of the aforementioned pig breeds, 60 pigs will be selected for the study.</td></tr>
<tr><td>Commitment</td><td colspan="3">I hereby commit to conducting research in full compliance with the National Standard Guidelines for the Ethical Review of Laboratory Animal Welfare (GB/T 35892-2018) throughout the project implementation. We will strive to minimize pain, distress, and discomfort in animals to the greatest extent possible through strict adherence to ethical protocols, thereby safeguarding animal rights and welfare.
Sign: Xinmiao He</td></tr>
<tr><td>Review Comments</td><td colspan="3">The design and protocol of this study fully consider safety and fairness, taking into account the replacement, reduction, and refinement of experimental animals, to protect the rights of animals to the greatest extent.
1/12/2024</td></tr>
</table>
